# ForeSpeed: A Real-World Video Dataset of CCTV Cameras with Different Settings for Vehicle Speed Estimation

**DOI:** 10.3390/jimaging12100488

**Published:** 2026-10-06

**Authors:** Massimo Iuliani, Blake Sawyer, Marco Fontani, David Spreadborough, Martino Jerian

**Affiliations:** 1 Amped Software, 34149 Trieste, Italy; marco.fontani@ampedsoftware.com (M.F.); david.spreadborough@ampedsoftware.com (D.S.); martino.jerian@ampedsoftware.com (M.J.); 2 Amped Software USA, Brooklyn, NY 11201, USA; blake.sawyer@ampedsoftware.com

**Keywords:** speed estimation, forensic dataset, collision investigation, video forensics

## Abstract

The need to estimate the speed of road vehicles has become increasingly important in the field of video forensics, particularly with the widespread deployment of CCTV cameras worldwide. Despite the development of various approaches, the accuracy of forensic speed estimation from real-world footage remains highly dependent on several factors, including camera specifications, acquisition methods, spatial and temporal resolution, compression methods, and scene perspective, all of which can significantly influence performance. In this paper, we introduce ForeSpeed, a comprehensive dataset designed to support the evaluation of speed estimation techniques in real-world scenarios using CCTV footage. The dataset includes recordings of 14 vehicle passes at known speeds, captured by three digital and three analog cameras from two distinct perspectives. Real-world road metrics are provided to enable the restoration of the scene geometry. Videos were exported with multiple compression levels and settings to simulate real-world scenarios in which export procedures are not always performed according to forensic standards. Overall, ForeSpeed includes a collection of 322 videos. As a case study, we employed the ForeSpeed dataset to benchmark a speed estimation algorithm available in a commercial product (Amped FIVE). The results demonstrate that while the method reliably estimates average speed across various conditions, the coverage rate and the estimated error range can be significantly affected by perspective. Furthermore, having higher spatial or temporal resolution alone does not guarantee better reliability of the estimates. The ForeSpeed dataset is publicly available to the forensic community, with the aim of facilitating the evaluation of current methodologies and inspiring the development of new, robust solutions tailored to collision investigation and forensic incident analysis.

## 1. Introduction

The speed of a vehicle prior to a collision is a critical factor in reconstructing the dynamics of an accident and assigning responsibility among the involved parties. In many forensic cases, however, vehicles are recorded by general-purpose closed-circuit television (CCTV) cameras rather than dedicated traffic monitoring systems. This results in practical limitations such as poor camera positioning, low or variable frame rates, and the need to manage proprietary formats. These limitations pose significant challenges for accurate forensic analysis and require an assessment of how these real-world degrading factors impact the speed estimation prior to a collision.

In the last decades, several methods have been designed for image-based speed estimation targeting various application fields [1], including those in which speed estimation serves as a component of a broader system [2]. Several approaches are designed to operate in controlled environments (e.g., traffic analysis), where automated speed estimation is attempted [3,4,5,6,7,8]. Many techniques leverage geometric cues such as reverse projection [9,10,11], or other perspective-based properties that can be applied under certain constraints, e.g., when the vehicle has a longitudinal trajectory relative to the camera [12] or the plate structure can be used to assess the vehicle speed [13,14]. Motion analysis across consecutive frames is another common strategy, exploiting visual-related features to gather information about a vehicle speed [15,16,17]. Previous studies have also shown that speed detection accuracy can be improved with the availability of multiple cameras or stereo images [18,19]. Other methods exploit projective properties such as the cross-ratio [20]; some are designed specifically for forensic CCTV scenarios [21], including challenging conditions such as nighttime scenes and uncertain visual anchors [22,23]. Recent advances in deep learning led to the development of data driven approaches to estimate speed in different conditions [24,25,26]. It is worth noting that in forensic scenarios, the methods used for the analysis are governed by several strict best practices. Several deep-learning architectures, by nature, may be difficult to interpret and the opacity of these models may impede the reproducibility, auditability, validation, error characterization, and independent scrutiny expected for forensic methods [27,28]. These limitations can create challenges when demonstrating alignment with digital-evidence standards and applicable legal or regulatory frameworks, including ISO/IEC 27037 [29], the Daubert standard, the EU AI Act, and the UK Forensic Science Regulator’s Notification 02-2024 [30]. These constraints place deep learning techniques in a challenging position regarding their practical usability in forensic environments. Moreover, such methods are typically optimized for large-scale traffic monitoring rather than the forensic analysis of a single vehicle captured by a consumer-grade camera with proprietary settings, non-standard formats, and arbitrary mounting angles. To sum up, although numerous methods exist, applying them in real-world scenarios can be challenging, since surveillance cameras are typically not designed for precise speed analysis. Their settings, such as low spatial and temporal resolutions, intrinsically limit the accuracy of assessments. Additionally, camera lenses can introduce strong image distortion that must be corrected prior to the estimate. Other complications include severe perspective angles or distant viewpoints, as well as compression artifacts that can introduce uncertainty in determining the exact vehicle position.

Amidst this complex interplay of variables, the forensic practitioner must estimate the vehicle’s speed with the highest possible accuracy, bearing full responsibility for the chosen method or line of reasoning applied in the case. From this standpoint, the proper acquisition of video evidence represents the critical first step in preserving both recorded events and associated metadata [31,32]. Subsequently, the collision investigator must apply an appropriate speed estimation technique, while also evaluating the reliability of the result within the specific case context, which is often far from ideal [33]. Indeed, officers frequently encounter material that has not been acquired in compliance with best practices [34], especially when it comes to the proper export of video files.

A number of datasets have been introduced in the literature to evaluate speed estimation methods. In some cases, these datasets focus on specific imaging devices, such as dashcams [35]. However, there remains limited experimental validation for scenarios that reflect the uncontrolled conditions typical of forensic investigations using CCTV footage. One of the most widely used datasets for traffic and vehicle speed detection is the AI City Challenge [4,36]. It provides a few video tracks recorded in normal traffic situations, such as highways or intersections. Although the scene was captured with a very high meter-to-pixel ratio, the videos were captured with a high frame rate, and there is no variety of camera models. Similarly, BrnoCompSpeed [37] is a dataset that focuses on camera calibration for traffic analysis and visual speed measurements from a single monocular camera. The dataset contains 18 videos at a resolution of 1920 × 1080 pixels at 50 fps, including more than 20,000 tagged vehicles. However, the videos were captured using three cameras (Panasonic HC-X920, Panasonic HDC-SD90, and Sony Handycam HDR-PJ410) whose specifications differ substantially from those of typical CCTV cameras. Another dataset provides hours of video recorded under varying weather conditions on urban roads. Although diverse weather conditions can be useful to assess their impact on the task, only a single camera is available [38]. A more forensic-oriented effort can be found in [39], which explores speed estimation using dashcam videos recorded in diverse road environments. In this work, the author also considered some challenging scenarios, including working with re-recorded and re-encoded videos, which may cause alterations of playback speed and time resolution. However, no CCTV cameras are included in the dataset.

In this paper, we introduce ForeSpeed, a real-world video dataset of CCTV cameras with different settings for the analysis and testing of vehicle speed estimation methods. The dataset consists of video recordings of a vehicle traveling at known speeds, captured by three digital and three analog cameras connected to three different digital video recorders (DVRs) with varying technical specifications, including differences in resolution and encoding schemes. The vehicle was filmed from two distinct viewpoints: one in which it moves parallel to the camera, and another where it approaches or recedes from the camera with stronger perspective while maintaining constant speed.

The dataset is specifically designed to determine whether and how individual parameters may influence the performance of speed estimation methods. As a case study, we applied ForeSpeed to benchmark one such method. Our analysis highlights how the coverage rate and the uncertainty range are affected by scene perspective and by spatial and temporal resolution.

## 2. ForeSpeed: A Dataset for Forensic Speed Estimation

The proposed dataset serves as a benchmark for evaluating the impact of various image acquisition factors on the accuracy of speed estimation. These factors include scene perspectives, camera/DVR models, their technical specifications, spatial and temporal resolution. Scene perspective plays a critical role in both point selection and meter-to-pixel ratio, that is, the portion of the road segment represented by each pixel. When the road is viewed from a low-angle perspective (where its direction is nearly parallel to the image plane), the meter-to-pixel ratio tends to remain relatively consistent along the vehicle’s path. In contrast, a strong perspective, where the road direction forms a wide angle with the image plane, introduces significant variation in the vehicle’s distance from the camera as it moves. This change has a notable impact on pixel interpretation: as the vehicle moves away from the camera, the meter-to-pixel ratio increases rapidly, and identifying the contact points between the wheels and the road becomes increasingly uncertain.

Different lens models and encoding schemes can produce markedly different digital representations of a video sequence. Lens distortion may cause straight lines to appear curved, compromising geometric measurements, while heavy compression can blur edges, introducing a laundering effect that complicates accurate point selection. These factors can significantly influence the speed estimation process and must be appropriately addressed: lens distortion should be corrected, and uncertainty in point selection should be evaluated on a case-by-case basis by the forensic analyst. In addition, the level of detail, when recording the same scene, varies substantially depending on the camera’s spatial and temporal resolution. For example, low-resolution imagery and variable frame rate acquisition can substantially degrade the precision of the speed estimate.

To account for such variability, we employed both digital and analog cameras, the latter connected to digital video recorders, as in the following:

- Three digital cameras, namely Eufy (T8441), Kasa (KC105), Ring (2nd generation);
- Two 1080p analog cameras, each connected to three DVRs, namely Anran, Lorex (D862A8-Z), Swann (DVR8-4680WRN);
- A 4K analog camera connected to the Lorex DVR (Anran and Swann did not support such a resolution).

Both Lorex and Swann allowed the acquisition of two different streams, namely Main and Sub, both reflected in the dataset. Moreover, when possible, videos were exported in multiple formats (.avi and .mp4). A diagram of the system used is provided in Figure 1. Note that, for simplicity, a label was assigned to each physical camera (from CAM-A to CAM-F).

The above setup was used to capture a vehicle traveling on a road at a known speed. All cameras were mounted on a wooden platform, enabling them to capture the scene from approximately the same position and with consistent rotation angles relative to the road plane. The platform was repositioned to record from two distinct perspectives: (i) a low perspective view, where the vehicle travels nearly parallel to the image plane; and (ii) a strong perspective view, where the road direction forms a wider angle relative to the image plane. Figure 2a and Figure 2b present examples of the same scene captured under low and strong perspective view, respectively.

More specifically, we collected 6 and 8 passes for the low and strong perspective cases, respectively. The vehicle, a 2020 Ford F-150 4x2 Supercrew (Overall, the vehicle is 231.9 inches, with a wheelbase of 145 inches. Technical specs of the vehicle can be found on https://media.ford.com/content/dam/fordmedia/North%20America/US/product/2020/f150/2020-F150-TechSpecs.pdf, accessed on 2 August 2026.), passes at 30 mph. The exact speed of the passes was checked through the following protocol: first, the vehicle’s onboard speedometer was independently validated using a high-precision external laser speed sensor to establish a reliable baseline; second, for every pass, a continuous video was recorded inside the vehicle cabin, capturing the real-time readouts of both the digital speedometer and an independent GPS-based speed application (see an example in Figure 3); finally, the vehicle speedometer and the external application’s reading were cross-checked when the vehicle passed the CCTV cameras. The speed of each pass is reported in Table 1.

We reported the speed values as integer since this was the sensitivity of both the speedometer and the external app. Each pass was simultaneously acquired with all available cameras and stored in multiple formats, when possible. Furthermore, we collected measurements from the road that can be used as a reference for estimating the vehicle speed. We used two different references in the videos captured under low and strong perspective views respectively. They are shown in Figure 4.

Since ForeSpeed is designed to resemble real-world scenarios, we exported each video in various formats and settings, based on the options available on each camera. The full list of available camera settings and specifications is reported in Table 2. We also report whether each video has a variable or constant frame rate. This classification was based on presentation timestamps, where available. For videos without timestamps, the frame type classification was inferred qualitatively through visual inspection of irregularities in the vehicle’s apparent motion across successive frames. We added an asterisk (*) to these cases to highlight this limitation as visual inspection alone cannot establish frame-rate variability, and no quantitative frame-timing data were available to verify the classification. Other technical details are available in Appendix A.

Each row of the table represents a video acquisition pipeline, involving a specific camera, a DVR (for analog cameras), and some export settings. More specifically, the Model column defines the model of the DVR or the digital camera; the Camera column defines the physical camera used, the Channel column defines the input channel of the DVR to which the camera is connected (set to ch01 for digital cameras). The fps column reports the frames per second. CFR and VFR stand for constant and variable frame rate, respectively. For each video, we report the acquisition camera, the spatial resolution, the observed codec, the storage format, the frame rate, and the type of frame rate (constant or variable).

As a result of the data and settings variability, the dataset exhibits broad diversity across key spatial and temporal parameters:

- Frame Rate: 132(41%) videos with 10 FPS or less; 190(59%) videos with more than 10 FPS;
- Frame Rate Type: 224(69.6%) constant frame rate videos and 98(30.4%) variable frame rate videos;
- Resolution: 168(52.2%) HD videos, 112(34.8%) lower resolution videos, and 42(13%) higher resolution videos.

All of the files within the dataset retain their original exported timing data. It is worth noting that, in the case of the Swann exports, this consists of three different frame timing sources (see Figure 5): (i) stream presentation timestamp, (ii) proprietary data timestamp, (iii) pixel-embedded timestamp. The availability of these data enables the evaluation of the impact of timing source selection and timing uncertainty.

## 3. 2D Speed Estimation

In this section, we summarize the speed estimation algorithm implemented in Amped FIVE that will be evaluated using ForeSpeed.

As an initial processing step, any optical distortion in the image must be corrected. This correction relies on the principle that straight lines in the real world should also appear straight in the 2D image. The distortion can be estimated by manually selecting the curved lines in the image that are expected to be straight once the distortion has been corrected [40]. We report an original camera frame and the corresponding frame after lens-distortion correction in Figure 6a and Figure 6b, respectively.

Under the assumption of a flat road, the image coordinates are transformed into real-world ground coordinates by the use of a planar homography that maps the image points of the street on its rectified top-down view [41]. In this new warped image, parallel lane markings will now appear completely parallel and pixel distance becomes linearly proportional to physical distances. Since the transformation has 8 degrees of freedom, it can be estimated through four coplanar points (i.e., the four corners of a rectangular object on the road). Eventually, the remaining ambiguity of the scale factor is solved by providing the length of the sides of a known rectangular shape on the road.

We report an example of a grid selection and the aerial image obtained by applying reverse projection on the selected grid in Figure 7a and Figure 7b, respectively.

The vehicle’s path can be defined as a sequence of contact points between the tires and the road. When defining the *i*-th contact point, the user can also set a pixel uncertainty of $m_{i}$ pixels, defining an error range of $\left(2m_{i}+1\right)\times \left(2m_{i}+1\right)$ pixels in which the actual contact point is expected to be. An example of selected vehicle’s path and contact point uncertainty region is shown in Figure 8a and Figure 8b, respectively. The geometric correction of the image yields the rectified contact points and uncertainty regions.

Formally, we can define the rectified path as a sequence of rectified contact points (${\bar{x}}_{i},t_{i}$), where ${\bar{x}}_{i}$ are the rectified spatial coordinates and $t_{i}$ are the temporal coordinates for each frame *i*, with i=0,…,N. The path defines a trajectory of length *d*, consisting of *N* segments of length $d_{1},\ldots ,d_{N}$, where $d_{i}$ is the distance between ${\bar{x}}_{i-1}$ and ${\bar{x}}_{i}$ (see Figure 9).

In Figure 10, the distance $d_{i}$ (blue arrow) is defined as the distance between the two rectified contact points (the two blue squares). We denote by $d_{i}^{\left(max\right)}$ and $d_{i}^{\left(min\right)}$ the maximum and minimum distances achievable by connecting points of the first rectified uncertainty region to points of the second rectified uncertainty region. In the figure, we visually show the maximum distance $d_{i}^{\left(max\right)}$ (the orange segment) and the minimum distance $d_{i}^{\left(min\right)}$ (the green segment) for that case.

Formally, we can define the error $\Delta d_{i}$ as

(1) $$\Delta d_{i}:=max\left\{d_{i}^{\left(max\right)}-d_{i},d_{i}-d_{i}^{\left(min\right)}\right\}.$$

In the end, a conservative estimate of the overall distance error can be obtained by summing the distance error on each segment:

(2) $$\Delta d=\sum_{j=1}^{N}\Delta d_{j},\;with\;d=\sum_{j=1}^{N}d_{j}.$$

The time uncertainty can be computed differently since it is mainly affected by $t_{0}$ and $t_{N}$. For simplicity, we assume that $\Delta t_{0}$ = $\Delta t_{N}$ = Δt. The CCTV timing source can often be obtained from the video’s presentation timestamps (PTS), or either from the CCTV data timestamp or pixel-embedded timestamp allocated to the video frame that represents date and time. All of these, though, require evaluation to ensure integrity, authenticity, and reliability. Timing and then the duration of each frame can also be calculated from the number of frames and duration; however, this will be an average and will not take variability into account. An external time reference, e.g., a lightboard, can be exploited to assist in establishing variability and aid in the identification of the most reliable timing source and the determination of the uncertainty associated with individual frames [42].

In the most common cases, under the assumption of a straight path, the average speed of a vehicle can be computed on a simplified model with two points only: the starting point (${\bar{x}}_{0},t_{0}$) and the final point (${\bar{x}}_{N},t_{N}$). The average speed can be computed as

(3) $$v=\frac{d}{t_{N}-t_{0}}$$

with relative distance ($\epsilon_{d}$) and time ($\epsilon_{t}$) errors   

(4) $$\epsilon_{d}=\frac{\Delta d}{d}\;\epsilon_{t}=\frac{2\cdot \Delta t}{t_{N}-t_{0}}.$$

Then, the relative speed error can be obtained as $\epsilon_{v}=\epsilon_{d}+\epsilon_{t}$ and the absolute speed error can be obtained as

(5) $$\Delta v=\epsilon_{v}\cdot v.$$

The method outputs the average speed *v* and the uncertainty range Δv, resulting in an estimated speed range [v−Δv,v+Δv]. Note that the above method, contrary to the cross-ratio approach [20,21], allows speed computation even when the vehicle is turning since the user can define a segmented path (by choosing N>1). In this case, the error component can be estimated on each path segment $d_{i}$ and the aggregated error can be obtained by summing all the error components. This is an intentionally conservative choice that provides an upper bound on the total error. Indeed, the error associated with one path segment generally influences that of an adjacent segment since they share a contact point. From this perspective, consecutive path segments exhibit a slight correlation, and the error model could potentially be refined to account for this coupling. However, this goes beyond the scope of this paper.

In the Algorithm 1, we report the pseudocode for estimating the average speed and the uncertainty range:

### Case Study: NFI Collaborative Exercise

The described speed estimation method was used in the “Collaborative Exercise of Speed from Video 2023”, proposed by the Netherlands Forensic Institute and distributed among the ENFSI Digital Imaging Working Group and Road Accident Analysis Working Group. Given a questioned video file, examiners were asked to determine the average speed of a car over a given trajectory. The questioned video had good quality, with a spatial resolution of 3840×2160 and 15 frames per second. Car characteristics, real-world measurements, and other reference recordings were also provided. In this specific case, the path is composed of two road segments on different planes connected by a bump. For that reason, the speed was estimated with two measurements, before and after the bump, respectively (namely, Measure 1 and Measure 2). In both cases, grid measurements from the road were used to get real-world distances. We achieved 82.5±8.6 and 84.4±8.8 Km/h respectively, over a real car speed of 81 Km/h, thus overestimating the average speed of 1.9% and 4.2% respectively (To ensure consistency with the evaluation conducted in Europe, the results are presented in km/h.).
**Algorithm 1** Pseudocode for estimating the average speed and the uncertainty range of the tested method1:Correct the optical distortion in every frame2:Estimate the planar homography H from the selected road-plane reference points for top-down view and add reference measures3:**for** i←0 to 1 **do**4:    Select the tire–road contact point ${\bar{p}}_{i}\leftarrow \left(u_{i},v_{i}\right)$ in frame $I_{i}$ with timestamp $t_{i}$5:    Define corresponding pixel-domain uncertainty $m_{i}$ and the corresponding region$$P_{i}\leftarrow \left\{\left(u,v\right):|u-u_{i}|\leq m_{i},\;\left|v-v_{i}\right|\leq m_{i}\right\}$$6:    Rectify the selected contact point:$${\bar{x}}_{i}\leftarrow \mathrm{PROJ}\left(H,{\bar{p}}_{i}\right)$$7:    Rectify the corresponding uncertainty region:$$X_{i}\leftarrow \left\{\mathrm{PROJ}\left(H,\bar{p}\right):\bar{p}\in P_{i}\right\}$$8:**end for**9:Compute path length $d=d_{1}\leftarrow {∥{\bar{x}}_{1}-{\bar{x}}_{0}∥}_{2}$10:Compute the average speed:$$v\leftarrow \frac{d}{t_{1}-t_{0}}$$11:Compute $d_{1}^{\left(\min\right)}$ and $d_{1}^{\left(\max\right)}$ on the rectified uncertainty regions $X_{0}$, $X_{1}$12:Compute distance uncertainty$$\Delta d=\Delta d_{1}\leftarrow max\left\{d_{1}^{\left(max\right)}-d_{1},d_{1}-d_{1}^{\left(min\right)}\right\}$$13:Compute the relative distance and timing uncertainties:$$\epsilon_{d}\leftarrow \frac{\Delta d}{d},\;\epsilon_{t}\leftarrow \frac{2\Delta t}{t_{1}-t_{0}}$$14:Compute the relative and absolute speed uncertainties:$$\epsilon_{v}\leftarrow \epsilon_{d}+\epsilon_{t},\;\Delta v\leftarrow \epsilon_{v}v$$15:**return** *v* and [v−Δv,v+Δv]

It is worth analyzing the uncertainty estimation in this specific case, as each car’s trajectory includes only five frames. Short distances and time intervals inherently increase the relative speed error (see Equation (4)), regardless of the method used. To highlight the significance of this factor, Table 3 reports the absolute speed error obtained in the two measurements, based on the length of the considered path. The first column shows the evaluated path length (for example, d1 + d2 indicates that speed is computed over the distance between frames 1 and 3). As expected, the more frames included in the computation, the lower the resulting uncertainty and the lowest uncertainty is observed when all available frames are used. This example demonstrates that limited information can significantly affect uncertainty, even when the estimated speed closely matches the actual value.

## 4. Experimental Results

The proposed dataset was employed to evaluate the reliability of the speed estimation method described in Section 3 across different scene perspectives, camera models, configurations, and export settings. Emulating a forensic scenario, the method was applied manually to each pass captured by each camera. Lens distortion correction and speed estimation were performed using the implementation provided by Amped FIVE.

An expert user selected the longest visible segment of the vehicle’s path for each video sequence. Videos for which contact points were not visible throughout the path were excluded from the analysis, thus assessing the vehicle speed on 269 video sequences (the list of the discarded videos that were not suitable for the method are reported within the provided repository, list of discarded files.txt). Uncertainty boxes were defined to ensure they contained the corresponding contact points (see Figure 8) and the frame capture time uncertainty was set to the default value Δt=5 ms. Note that this value refers to the timestamp uncertainty that is not strictly related to the frame rate. In Molnar et al. [42], calibrated LED lightboards were used as independent timing references to estimate the frame time uncertainty on a set of recording devices and cameras, achieving a mean discrepancy of 2.99 ms between nominal one-second timestamp intervals and the elapsed time measured by the lightboard.

In the scene, the known dimensions of the rectangular ground grid were manually selected to rectify the road plane and estimate real-world distances. For each video, we obtained an average speed *v* and an uncertainty range Δv (as defined in Equation (5)). Since the vehicle is moving in a straight line, we considered the longest visible path with two contact points only.

We then examined the error of the estimated average speed with respect to the ground-truth value. The raw error and the mean absolute error (MAE) for *v*, averaged across all the experiments, are −0.96 and 1.25 mph respectively, with a coverage of 255 over 269 valid estimates (94.8%) in which the ground-truth speed falls in the estimated range. The observed mean signed error is compatible with a systematic offset in the reference speeds, but the absence of a sub-integer precision does not allow us to distinguish this explanation from a systematic contribution of the estimation procedure, or a combination of both. When splitting the results by scene perspective (low vs. strong), the MAE remains comparable between the two cases (1.31 and 1.22 mph for low and strong perspective, respectively). However, under low perspective, the mean uncertainty range is 3.21 mph with a coverage of 88.8%, whereas under strong perspective we observe a mean uncertainty range of 8.31 mph and a coverage of 97.8%. In summary, a strong perspective yields higher coverage, at the cost of a wider uncertainty range (see Table 4). A reasonable explanation for the wider uncertainty range is that the pixel-to-meter ratio changes significantly across scenes with strong perspective as the vehicle moves farther from the camera thus resulting in wider selected error ranges for contact points. If we split the results by frame rate type (constant vs. variable frame rate), we note that the method achieves almost full coverage on variable frame rate videos and the MAE remains nearly identical across both groups (see Table 5).

A reasonable explanation is that video files were acquired in their original formats without re-encoding, in accordance with best forensic practices. This allowed us to retrieve the original presentation timestamps (PTS), which were used to compute a precise time delta between the initial and final frames.

We also reported a performance breakdown based on spatial and temporal resolution (see Table 6).

The results suggest that spatial resolution alone is insufficient to guarantee higher coverage. In this dataset, lower resolution actually yielded a higher coverage rate than ultra-high resolution. On this point, it is worth noting that the dataset contains high-resolution videos with very low frame rates (e.g., LOREX CAM-C at 3840 × 2160 pixels but only 7 FPS), which can strongly affect these outcomes. Indeed, when splitting the results between low and high FPS (see Table 7), the results support that a higher frame rate leads to higher coverage although it is linked to wider error ranges. The codec and container breakdowns are reported in Table 8, splitting H.264 and HEVC for codecs, and AVI and MP4 for containers. The results do not reveal marked differences in the accuracy or coverage based on format properties.

Finally, we analyzed a breakdown of the results based on camera/DVR models. The results, reported in Table 9, highlight that the coverage loss is not equally distributed across all cameras, but is primarily concentrated in the LOREX model. A deeper check reveals that the LOREX – CAM-C configuration has a coverage rate of 71.4% (30/42), accounting for 12 out of the 14 total failures in the entire dataset. The problem seems to be associated with the physical CAM-C as the method performs well when LOREX exploits a different physical camera.

We also examined the frequency with which the estimated speed either exactly matches the true speed or falls within small error margins (±1, ±2 mph). These results are summarized in Table 10. Notably, in 85% of cases, the estimated speed deviates from the true value within 2 mph, regardless of perspective.

We report the raw speed estimation errors for all passes in Table 11. Their histograms are reported in Figure 11a and Figure 11b, for the low and strong perspective views, respectively. Similarly, the uncertainty ranges Δv are summarized in Figure 12a,b. Although the validation was conducted by a single user and thus could not account for potential operator bias, these results show the effectiveness of the proposed dataset in revealing the behavior of the analyzed speed estimation method.

## 5. Conclusions

We introduced ForeSpeed, a real-world dataset of 322 CCTV videos. The dataset is designed to evaluate the effectiveness of speed estimation methods using footage captured under varying acquisition conditions, including different scene perspectives and a range of spatial and temporal camera resolutions. To resemble real scenarios, each video was exported in various formats and with various settings, based on the options available on each camera. As a case study, we employed ForeSpeed to assess the performance of a speed estimation method manually applied to the video sequences. The results breakdown showed how the method’s performance was affected by the main parameters: perspective angle, frame rate, frame-rate type, spatial resolution, and acquisition-system type. The method achieved a mean absolute error of 1.25 mph and an empirical coverage of 255/269 (94.8%) relative to the recorded reference speeds. Coverage was lower for the low-perspective sequences (88.8%) than for the strong-perspective sequences (97.8%), while the latter had a wider mean uncertainty range (8.31 versus 3.21 mph). Interestingly, having higher spatial or temporal resolution alone does not guarantee better reliability of the estimates in terms of mean absolute error and coverage rate.

ForeSpeed provides a valuable resource for exploring the limitations and specific characteristics of various speed estimation methods. Moreover, ForeSpeed includes synchronized recordings of the same scene from multiple camera viewpoints, enabling future research on multi-view approaches to speed estimation. In the future, this variability could be further extended by acquiring videos across a wider range of vehicle speeds or vehicle classes.

## Figures and Tables

**Figure 1:**
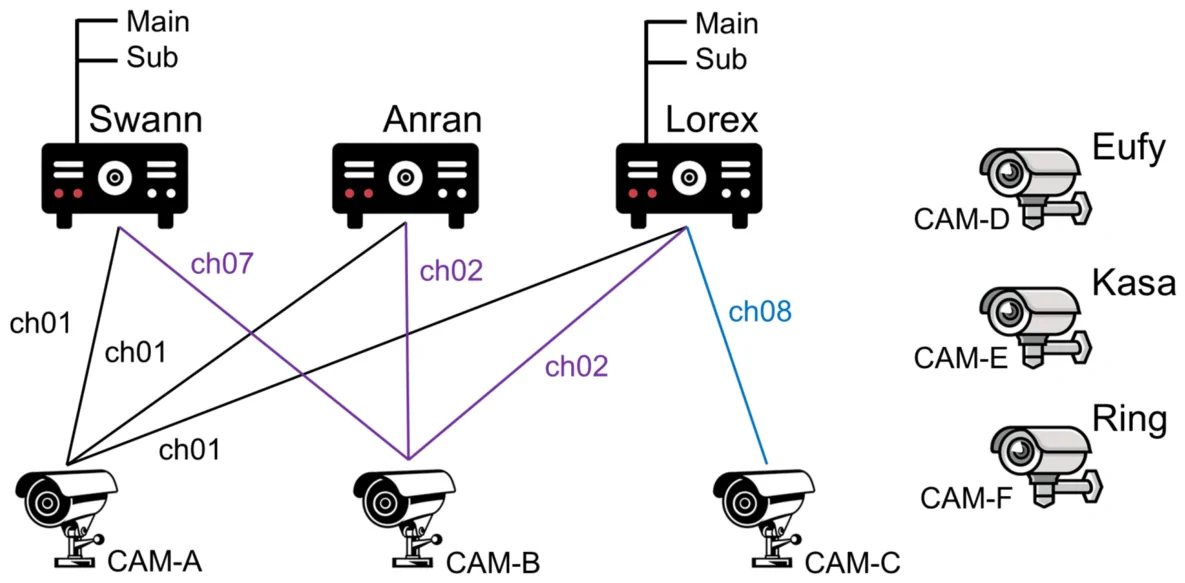
Dataset structure: three digital cameras and three analog cameras connected to different DVRs.

**Figure 2:**
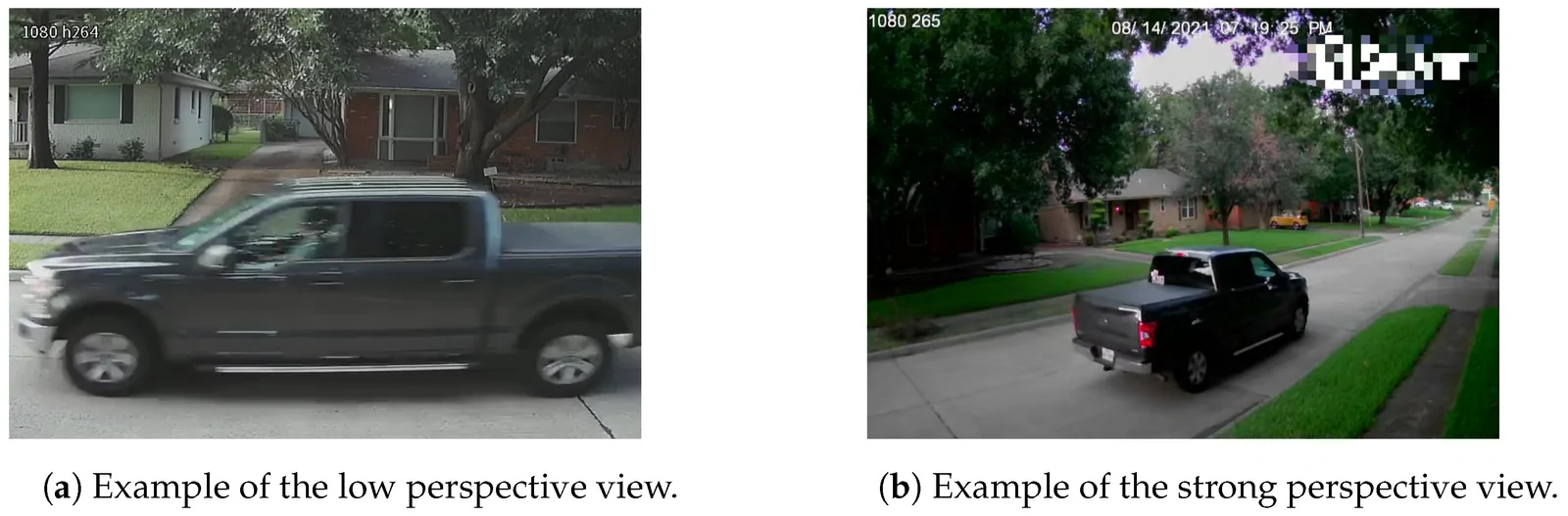
Example of the passes captured under two distinct perspectives: a low perspective view, where the vehicle travels nearly parallel to the image plane; and a strong perspective view, where the road direction forms a wider angle relative to the image plane. (**a**) Example of the low perspective view) and (**b**) Example of the strong perspective view.

**Figure 3:**
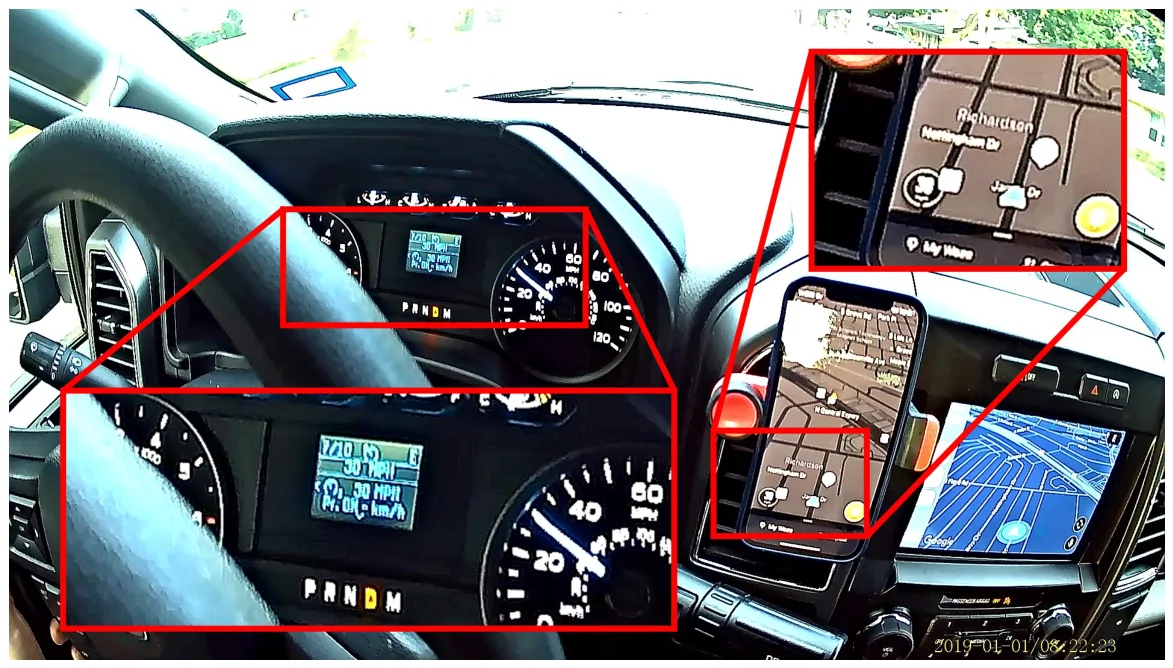
Speed visualization on the speedometer and an external GPS-based application.

**Figure 4:**
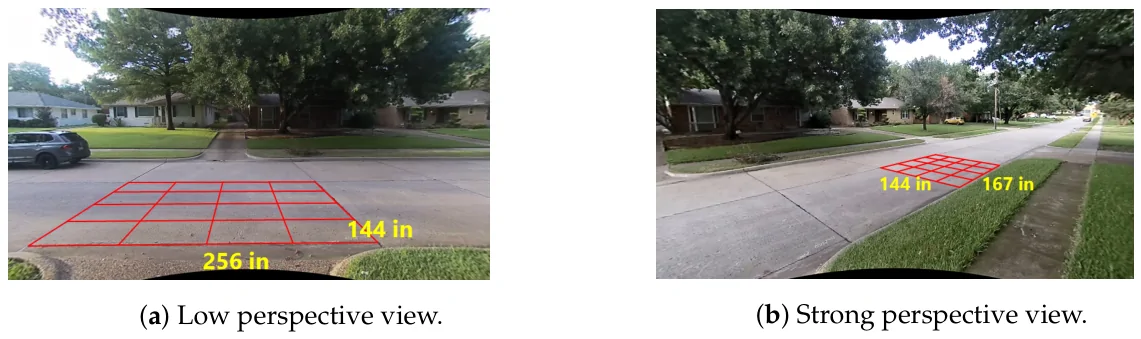
Collected measurements from the road that can be used to determine real-world coordinates from image coordinates. (**a**) Low perspective view and (**b**) Strong perspective view.

**Figure 5:**
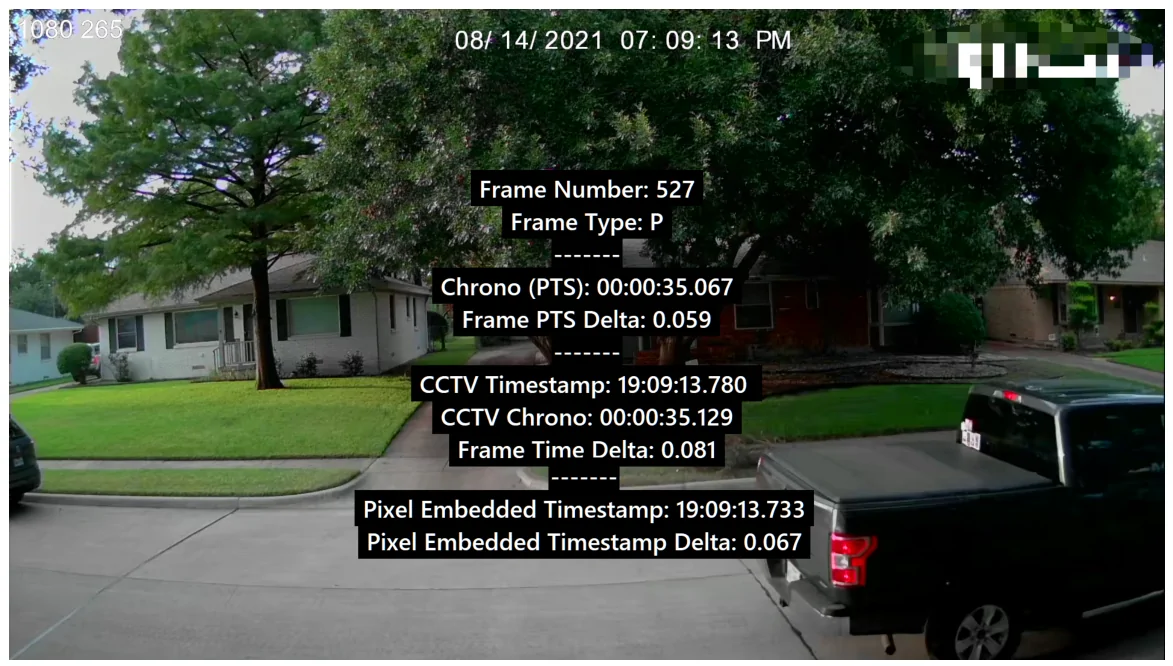
Example of multiple timing source available in Swann acquisition.

**Figure 6:**
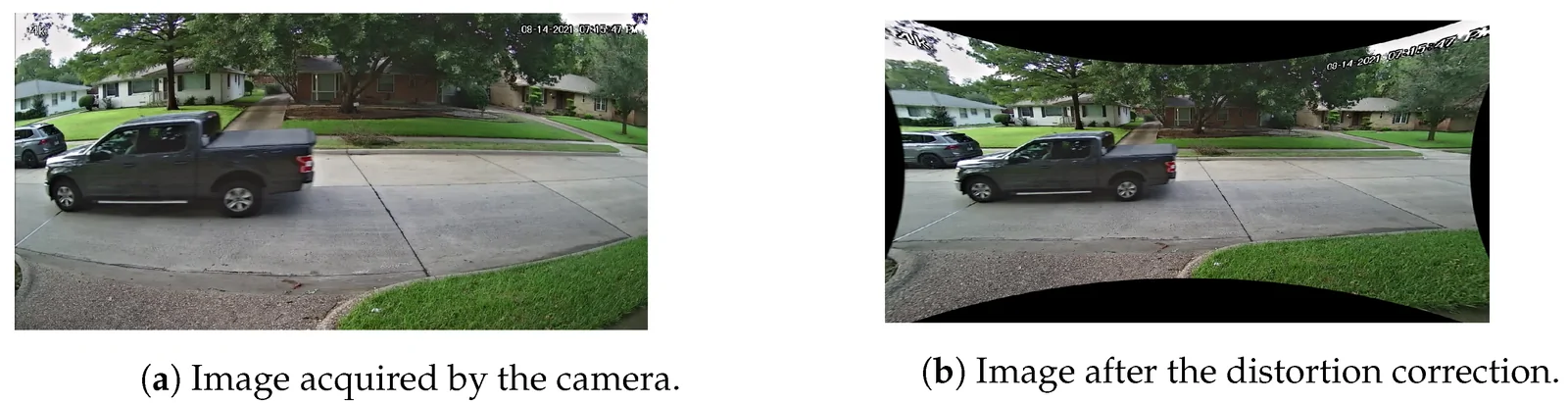
Example lens distortion correction. The distortion parameters are estimated by selecting the curved lines in the image that are expected to be straight once the distortion has been corrected. (**a**) Image acquired by the camera and (**b**) Image after the distortion correction.

**Figure 7:**
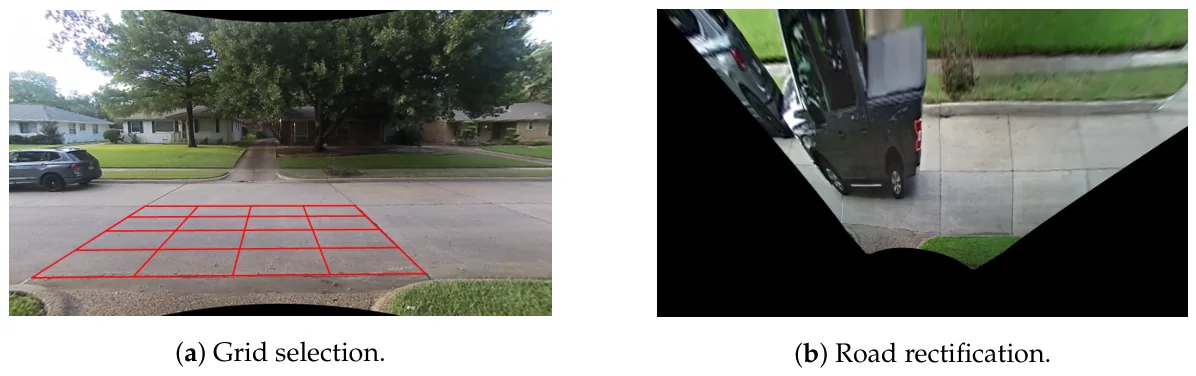
Examples of reference selection (**a**) and road rectification (**b**). In the new warped image, parallel lane markings become completely parallel and pixel distance becomes linearly proportional to physical distances. The transformation can be estimated through the four corners of a rectangular object on the road.

**Figure 8:**
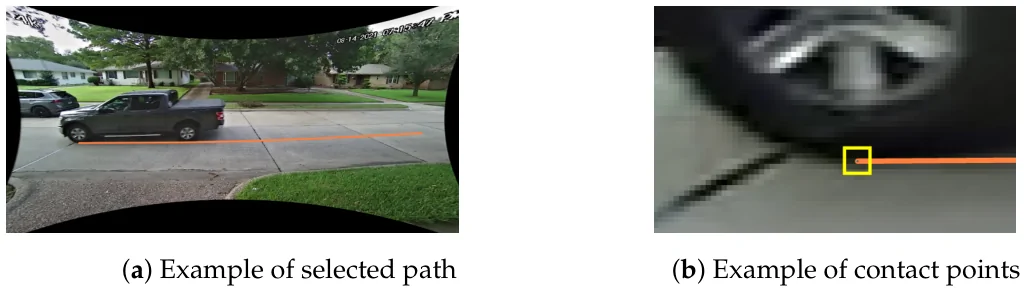
Examples of a rectified path (**a**) and the defined uncertainty region defined on a wheel contact point (**b**).

**Figure 9:**
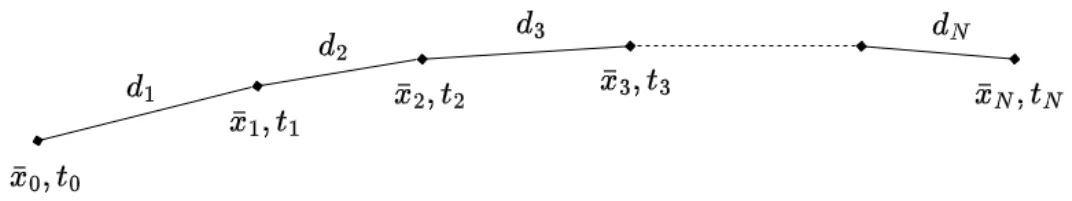
List of contact points defining the vehicle path.

**Figure 10:**
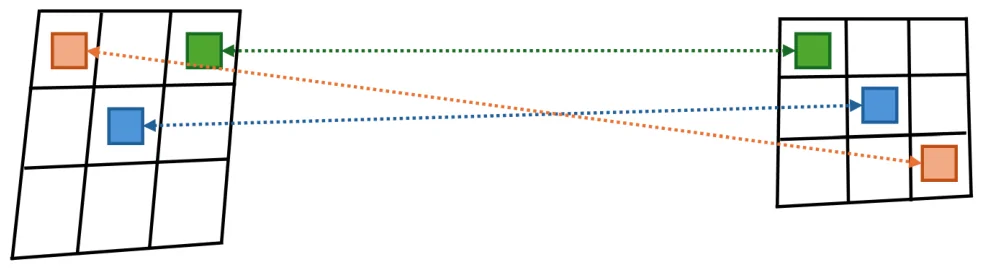
Example of rectified contact points in two consecutive positions with a pixel uncertainty m=1. The selected distance $d_{i}$ (the blue segment) lies between the maximum distance $d_{i}^{\left(max\right)}$ (the orange segment) and the minimum distance $d_{i}^{\left(min\right)}$ (the green segment).

**Figure 11:**
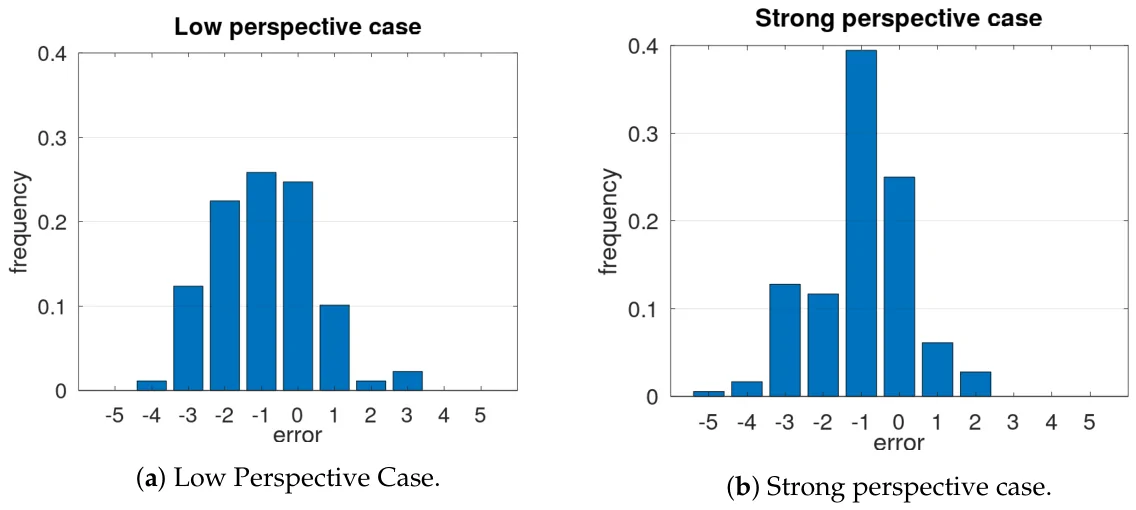
Histograms of the error between the estimated *v* and the vehicle real speed. (**a**) Low Perspective Case and (**b**) Strong Perspective Case.

**Figure 12:**
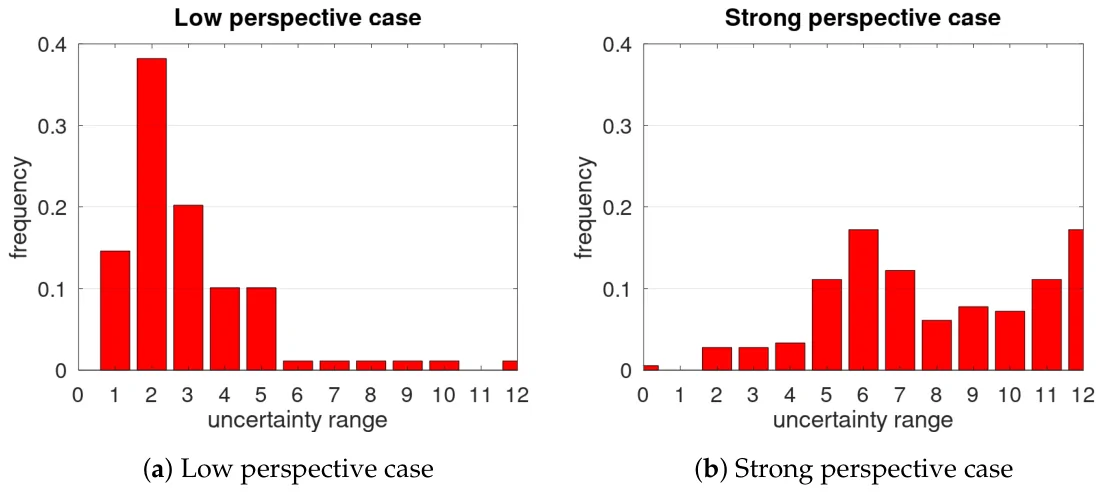
Histograms of Δv achieved in the speed estimates. Results are split by scene perspective (low vs. strong). (**a**) Low perspective case and (**b**) Strong perspective case.

**Table 1:** Ground-truth speed in mph for each pass. T1 and T2 stand for low and strong perspective, respectively.

Pass	GT Speed	Pass	GT Speed
T1P1	29	T2P1	30
T1P2	30	T2P2	30
T1P3	30	T2P3	30
T1P4	31	T2P4	30
T1P5	30	T2P5	30
T1P6	30	T2P6	31
		T2P7	30
		T2P8	29

**Table 2:** List of DVR/camera models, physical camera IDs, DVR connection channels, and format details (PTS stands for presentation timestamps availability, fps stands for frames per second, CFR and VFR stand for constant and variable frame rates respectively.). An asterisk (*) is added when the frame rate type was deduced through visual inspection of irregularities in the vehicle’s apparent motion across successive frames, as no quantitative frame-timing data were available to verify it.

Model	Camera	Channel	Format	Codec	Resolution	PTS	fps	fps Type
Anran	CAM-A	ch01	avi	hevc	1920 × 1080	No	14	VFR *
Anran	CAM-B	ch02	avi	hevc	1920 × 1080	No	14	VFR *
Eufy	CAM-D	ch01	mp4	h264	2304 × 1296	Yes	14.99	VFR
Kasa	CAM-E	ch01	mp4	h264	1920 × 1080	Yes	14.99	VFR
Lorex	CAM-A	ch01 main	avi	hevc	1920 × 1080	No	30	CFR *
Lorex	CAM-A	ch01 main	mp4	hevc	1920 × 1080	Yes	30	CFR
Lorex	CAM-A	ch01 sub	mp4	hevc	704 × 408	Yes	15	CFR
Lorex	CAM-B	ch02 main	avi	h264	1920 × 1080	No	30	CFR *
Lorex	CAM-B	ch02 main	mp4	h264	1920 × 1080	Yes	30	CFR
Lorex	CAM-B	ch02 sub	mp4	h264	960 × 480	Yes	10	CFR
Lorex	CAM-C	ch08 main	avi	hevc	3840 × 2160	No	7	CFR *
Lorex	CAM-C	ch08 main	mp4	hevc	3840 × 2160	Yes	7	CFR
Lorex	CAM-C	ch08 sub	mp4	h264	960 × 480	Yes	7	CFR
Ring	CAM-F	DevTools	mp4	h264	640 × 360	Yes	10	CFR
Ring	CAM-F	Download	mp4	h264	1920 × 1080	Yes	10	CFR
Swann	CAM-A	ch01 main	avi	hevc	1920 × 1080	No	15	CFR *
Swann	CAM-A	ch01 main	mp4	hevc	1920 × 1080	Yes	15.03	VFR
Swann	CAM-A	ch01 sub	avi	hevc	704 × 480	No	5	CFR *
Swann	CAM-A	ch01 sub	mp4	hevc	704 × 480	Yes	5	CFR
Swann	CAM-B	ch07 main	avi	h264	1920 × 1080	No	15	CFR *
Swann	CAM-B	ch07 main	mp4	h264	1920 × 1080	Yes	15.03	VFR
Swann	CAM-B	ch07 sub	avi	h264	704 × 480	No	5	CFR *
Swann	CAM-B	ch07 sub	mp4	h264	704 × 480	Yes	5.02	VFR

**Table 3:** Speed estimation achieved in the considered case study. Two independent estimations were performed (Measure 1 and 2) based on different real-world measurements. We assess the speed on different path lengths. For instance, $d_{1}+d_{2}$ refers to the speed computed on the first two distance segments (between frames 1 and 3).

Measure 1	*v*	Δv	Measure 2	*v*	Δv
$d_{1}$	82.0	26.0	$d_{1}$	81.8	31.8
$d_{1}+d_{2}$	82.6	14.1	$d_{1}+d_{2}$	84.2	16.7
$d_{1}+d_{2}+d_{3}$	83.0	10.3	$d_{1}+d_{2}+d_{3}$	84.5	11.4
$d_{1}+d_{2}+d_{3}+d_{4}$	82.5	8.6	$d_{1}+d_{2}+d_{3}+d_{4}$	84.4	8.8

**Table 4:** Results breakdown by perspective. Average statistics for estimated speed (in mph) are reported: Mean Absolute Error (MAE), the coverage rate, and the Mean Uncertainty Range Δv.

Case	MAE	Coverage	Unc. Range
Low Perspective	1.31	88.8% (79/89)	3.21
Strong Perspective	1.22	97.8% (176/180)	8.31

**Table 5:** Results breakdown by frame rate type (constant vs. variable).

Frame Rate Type	MAE	Coverage	Unc. Range
Constant	1.26	93.0% (172/185)	6.69
Variable	1.24	98.8% (83/84)	6.48

**Table 6:** Breakdown by spatial resolution (HD is 1920×1080).

Spatial Res.	MAE	Coverage	Unc. Range
Lower than HD	1.26	96.7% (89/92)	7.17
HD	1.19	98.5% (133/135)	7.03
Greater than HD	1.45	78.6% (33/42)	4.12

**Table 7:** Breakdown by temporal resolution (FPS denotes frames per second).

FPS	MAE	Coverage	Unc. Range
≤10	1.24	89.4% (101/113)	5.25
>10	1.26	98.7% (154/156)	7.62

**Table 8:** Descriptive breakdown by codec and container. MAE and mean uncertainty range are reported in mph.

Case	MAE	Coverage	Unc. Range
H.264	1.09	97.2% (139/143)	6.52
HEVC	1.44	92.1% (116/126)	6.75
AVI	1.16	95.0% (96/101)	6.33
MP4	1.31	94.6% (159/168)	6.80

**Table 9:** Breakdown by camera/DVR model.

Model	MAE	Coverage	Unc. Range
ANRAN	1.30	95.7% (22/23)	6.78
EUFY	1.57	100.0% (14/14)	6.71
KASA	1.29	100.0% (14/14)	6.36
LOREX	1.32	87.7% (93/106)	6.36
RING	1.21	100.0% (24/24)	8.33
SWANN	1.11	100.0% (88/88)	6.47

**Table 10:** The columns report the percentage of speed estimates within a specific error range. For instance, in the low perspective case, the absolute error is within 1 mph the actual speed in 54 tests (representing 61% of the cases).

Absolute Error Range	Exact Speed	±1 mph	±2 mph
Low perspective case	25% (22)	61% (54)	84% (75)
Strong perspective case	25% (45)	71% (127)	85% (153)

**Table 11:** Signed speed estimation errors (mph) for the 23 acquisition pipelines. T1 and T2 denote low- and strong-perspective conditions, respectively. The symbol × indicates an excluded sequence. Bold entries indicate cases in which the estimates fell out of the coverage interval.

			T1: Low Perspective						T2: Strong Perspective							
Model	Camera	Export	P1	P2	P3	P4	P5	P6	P1	P2	P3	P4	P5	P6	P7	P8
Anran	CAM-A	–	×	−2	−2	+1	×	+1	+1	−1	−3	0	0	−2	0	0
Anran	CAM-B	–	×	−4	×	−1	×	0	−2	−1	−2	−1	−1	−2	0	−3
Eufy	CAM-D	–	−3	−2	−2	−2	−3	−1	0	−1	−1	−1	0	−3	−1	−2
Kasa	CAM-E	–	−2	−3	−2	−2	−2	−2	0	+1	0	+1	+1	−2	0	0
Lorex	CAM-A	Main AVI	×	−2	×	−2	×	−1	×	−2	×	−1	−1	−3	0	−2
Lorex	CAM-A	Main MP4	×	−1	×	0	×	0	0	−2	−1	−2	−1	−4	0	−3
Lorex	CAM-A	Sub MP4	×	−2	×	−3	×	−1	−3	−1	−1	−1	−1	−3	−2	−2
Lorex	CAM-B	Main AVI	×	−1	×	−1	×	0	−1	−1	−1	−1	−1	−3	−1	−2
Lorex	CAM-B	Main MP4	×	−1	×	−1	×	0	0	−1	−1	0	−1	−3	−1	−1
Lorex	CAM-B	Sub MP4	×	−1	×	−1	×	0	−3	−1	0	−1	0	−3	0	−1
Lorex	CAM-C	Main AVI	+1	−1	+1	−3	3	−3	0	−1	0	0	+1	−3	−1	−1
Lorex	CAM-C	Main MP4	−3	0	+1	−3	+3	−3	0	0	+1	0	+1	−3	−1	−1
Lorex	CAM-C	Sub MP4	0	−1	0	−3	+2	−3	−1	0	−5	0	+2	−2	0	0
Ring	CAM-F	DevTools	×	−1	+1	0	+1	+0	−1	−1	−1	×	−2	−4	−1	−1
Ring	CAM-F	Download	×	−2	0	−2	−2	−1	+2	+2	2	×	+1	0	+1	0
Swann	CAM-A	Main AVI	×	−2	×	0	×	−2	−1	−1	−1	−1	−1	−3	−1	−2
Swann	CAM-A	Main MP4	×	−2	×	−2	×	0	−1	−1	−2	−1	−1	−3	−1	−1
Swann	CAM-A	Sub AVI	×	−1	×	−1	×	−1	+1	0	+1	0	+2	−2	0	−2
Swann	CAM-A	Sub MP4	×	−1	×	−1	×	0	−3	−1	−3	−2	−3	−4	−3	−2
Swann	CAM-B	Main AVI	×	0	×	0	×	+1	−1	0	−1	0	0	−3	−1	−1
Swann	CAM-B	Main MP4	×	0	×	−1	×	0	−1	0	−1	−1	0	−3	0	−1
Swann	CAM-B	Sub AVI	×	0	×	0	×	+1	0	−1	−1	0	0	−3	0	−1
Swann	CAM-B	Sub MP4	×	−1	×	−1	×	0	−1	−1	−1	0	−1	−3	−1	−1

## Data Availability

The ForeSpeed dataset presented in this study is publicly available and can be downloaded from GitHub at: https://github.com/ampedsoftware/forespeed (accessed on 2 August 2026).

## Appendix A. Dataset Structure

ForeSpeed comprises 322 videos of 14 physical passes recorded simultaneously by multiple cameras and subsequently exported using different streams and file formats. Since the dataset is designed for forensic assessment, we preserved the original video information that is relevant for the task, such as the presentation timestamps (PTSs) of variable frame rate videos that are stored in the video container. This led to some constraints in the file naming and splitting. The dataset is structured as follows:

- Six folders, each containing the data acquired by a CCTV camera model (e.g., Eufy).
- Each folder may contain multiple sub-folders if multiple cameras and export formats are available (e.g., Lorex/ch01/main/avi). These folders may include different CCTV cameras plugged into the same DVR, multiple quality streams that are available by the DVR, and multiple compression settings.
- Each sub-folder contains the 6 and 8 passes under the two considered perspectives. When possible, the video containing the pass is stored with the name T<x>P<y> -<camera name>-<details>, where T1 and T2 stand for low and strong perspective respectively and P identifies the pass (e.g., T1P5-Ring-6996443541347278144 stands for the pass 5 with a low perspective in the camera Ring).

Since each camera uses a different acquisition protocol, some exceptions occurred:

- Anran acquisition is split in four videos of 15 min each;
- For some cameras the video acquisition is automatically split. When the split occurs during the vehicle pass, two videos of the same pass are included in the dataset (e.g., Lorex/ch01/main/avi/T1P4).

The number of the test and the pass is physically shown in the video before the pass. The hierarchical structure of the dataset is fully described in the provided repository.

## References

[1] Fernández Llorca D., Hernández Martínez A., Garcia Daza I. (2021). Vision-based vehicle speed estimation: A survey. IET Intell. Transp. Syst..

[2] Xu X., Wu Q., Fan P., Wang K., Cheng N., Chen W., Letaief K.B. (2026). Velocity-adaptive access scheme for semantic-aware vehicular networks: Joint fairness and AoI optimization. IEEE Trans. Mob. Comput..

[3] Afifah F., Nasrin S., Mukit A. (2019). Vehicle speed estimation using image processing. J. Adv. Res. Appl. Mech..

[4] Huang T. (2018). Traffic speed estimation from surveillance video data. Proceedings of the IEEE Conference on Computer Vision and Pattern Recognition Workshops, Salt Lake City, UT, USA, 18–22 June 2018.

[5] Doğan S., Temiz M.S., Külür S. (2010). Real time speed estimation of moving vehicles from side view images from an uncalibrated video camera. Sensors.

[6] Celik T., Kusetogullari H. (2009). Solar-powered automated road surveillance system for speed violation detection. IEEE Trans. Ind. Electron..

[7] He Z., Liu Y., Ye X. (2007). Models of vehicle speeds measurement with a single camera. Proceedings of the 2007 International Conference on Computational Intelligence and Security Workshops (CISW 2007).

[8] Schoepflin T.N., Dailey D.J. (2003). Dynamic camera calibration of roadside traffic management cameras for vehicle speed estimation. IEEE Trans. Intell. Transp. Syst..

[9] Epstein B., Westlake B.G. (2019). Determination of vehicle speed from recorded video using reverse projection photogrammetry and file metadata. J. Forensic Sci..

[10] Edelman G., Bijhold J. (2010). Tracking people and cars using 3D modeling and CCTV. Forensic Sci. Int..

[11] Cathey F., Dailey D. (2005). A novel technique to dynamically measure vehicle speed using uncalibrated roadway cameras. Proceedings of the Intelligent Vehicles Symposium.

[12] Costa L.R., Rauen M.S., Fronza A.B. (2020). Car speed estimation based on image scale factor. Forensic Sci. Int..

[13] Luvizon D.C., Nassu B.T., Minetto R. (2014). Vehicle speed estimation by license plate detection and tracking. Proceedings of the 2014 IEEE International Conference on Acoustics, Speech and Signal Processing (ICASSP).

[14] Vakili E., Shoaran M., Sarmadi M.R. (2020). Single–camera vehicle speed measurement using the geometry of the imaging system. Multimed. Tools Appl..

[15] Lin L., Ramesh B., Xiang C. (2015). Biologically inspired composite vision system for multiple depth-of-field vehicle tracking and speed detection. Proceedings of the Computer Vision-ACCV 2014 Workshops, Singapore, Singapore, 1–2 November 2014.

[16] Lan J., Li J., Hu G., Ran B., Wang L. (2014). Vehicle speed measurement based on gray constraint optical flow algorithm. Optik.

[17] Xu Q., Li X., Wu M., Li B., Song X. (2014). A methodology of vehicle speed estimation based on optical flow. Proceedings of the 2014 IEEE International Conference on Service Operations and Logistics, and Informatics.

[18] Llorca D.F., Salinas C., Jimenez M., Parra I., Morcillo A., Izquierdo R., Lorenzo J., Sotelo M. (2016). Two-camera based accurate vehicle speed measurement using average speed at a fixed point. Proceedings of the 2016 IEEE 19th International Conference on Intelligent Transportation Systems (ITSC).

[19] El Bouziady A., Thami R.O.H., Ghogho M., Bourja O., El Fkihi S. (2018). Vehicle speed estimation using extracted SURF features from stereo images. Proceedings of the 2018 International Conference on Intelligent Systems and Computer Vision (ISCV).

[20] Han I. (2016). Car speed estimation based on cross-ratio using video data of car-mounted camera (black box). Forensic Sci. Int..

[21] Wong T., Tao C., Cheng Y., Wong K., Tam C. (2014). Application of cross-ratio in traffic accident reconstruction. Forensic Sci. Int..

[22] Yang W.C., Jiang J., Mao A., Su K.A., Chen C.H. (2024). The study on the estimation of vehicles speed using a dashboard camera. Multimed. Tools Appl..

[23] Choi Y., Yun Y., Jeon W.J., Kong S.H. (2023). A Novel Approach for Estimating Vehicle Speed in Nighttime Traffic Accidents from Daytime Video Information. Proceedings of the 2023 IEEE 26th International Conference on Intelligent Transportation Systems (ITSC).

[24] Kamil D.A., Harjoko A., Jo K.H. (2024). Vehicle speed estimation using consecutive frame approaches and deep image homography for image rectification on monocular videos. IEEE Access.

[25] Shanmugam P., Shanmugathai M., Keshari S., Ananth S.J., Sujatha S. (2025). Mask R-CNN-based Vehicle Speed Estimation for Enhancing Road Safety and Control. Proceedings of the 2025 5th International Conference on Soft Computing for Security Applications (ICSCSA).

[26] Choi Y., Kong S.H. (2026). Robust video-based vehicle speed estimation for occluded scenes for forensic analysis of traffic accidents. Multimed. Tools Appl..

[27] Holdren J.P., Lander E.S., Press W., Savitz M. (2016). Forensic Science in Criminal Courts: Ensuring Scientific Validity of Feature-Comparison Methods. President’s Committee of Advisors on Science and Technology. https://obamawhitehouse.archives.gov/sites/default/files/microsites/ostp/PCAST/pcast_forensic_science_report_final.pdf.

[28] State v. Swinton, 268 Conn. 781, 847 A.2d 921, 2004. Pages 829–830, Supreme Court of the State of Connecticut. https://law.justia.com/cases/connecticut/supreme-court/2004/268cr60.html.

[29] ISO/IEC (2012). Information Technology—Security Techniques—Guidelines for Identification, Collection, Acquisition and Preservation of Digital Evidence.

[30] Forensic Science Regulator Regulator’s Notification 02/2024: Estimating Vehicle Speed from Video Footage. Regulator’s Notification 02/2024, Home Office, UK Government, 2024. https://www.gov.uk/government/publications/regulators-notification-02-2024-estimating-vehicle-speed-from-video-footage.

[31] Lallie H.S. (2023). Dashcam forensic investigation guidelines. Forensic Sci. Int. Digit. Investig..

[32] Wooller A. Digital storage CCTV systems: A guide to the effectiveness and evidential usage of digital CCTV. Proceedings of the Institute of Traffic Accident Investigators (ITAI) International Conference, 7th, 2005.

[33] Hoogeboom B., Alberink I. (2010). Measurement uncertainty when estimating the velocity of an allegedly speeding vehicle from images. J. Forensic Sci..

[34] SWGDE (2024). Best Practices for Digital Forensic Video Analysis. https://www.swgde.org/wp-content/uploads/2024/04/2024-03-22-SWGDE-Best-Practices-for-Digital-Forensic-Video-Analysis-18-V-001-1.1.pdf.

[35] Lallie H.S. (2020). Dashcam forensics: A preliminary analysis of 7 dashcam devices. Forensic Sci. Int. Digit. Investig..

[36] Hua S., Kapoor M., Anastasiu D.C. (2018). Vehicle tracking and speed estimation from traffic videos. Proceedings of the IEEE Conference on Computer Vision and Pattern Recognition Workshops.

[37] Sochor J., Juránek R., Španhel J., Maršık L., Široky A., Herout A., Zemcık P. (2017). Brnocompspeed: Review of traffic camera calibration and comprehensive dataset for monocular speed measurement. arXiv.

[38] Luvizon D.C., Nassu B.T., Minetto R. (2016). A video-based system for vehicle speed measurement in urban roadways. IEEE Trans. Intell. Transp. Syst..

[39] Shim K.S., in Park N., Kim J.H., Jeon O.Y., Lee H. (2021). Vehicle speed measurement methodology robust to playback speed-manipulated video file. IEEE Access.

[40] De Haan G., Bellers E.B. (1998). Deinterlacing—An overview. Proc. IEEE.

[41] Jain A.K. (1989). Fundamentals of Digital Image Processing.

[42] Molnar B., Terpstra T., Voitel T. (2025). Accuracy of Timestamps in Digital and Network Video Recorders.

